# Perceptions of medical graduates and their workplace supervisors towards a medical school clinical audit program

**DOI:** 10.5116/ijme.592a.a936

**Published:** 2017-07-07

**Authors:** Stephanie Davis, Ilse O'Ferrall, Samuel Hoare, Bulsara Caroline, Donna B. Mak

**Affiliations:** 1 School of Medicine, Fremantle, University of Notre Dame Australia, Western Australia, Australia; 2 School of Nursing and Midwifery, University of Notre Dame, Australia

**Keywords:** Curriculum development, post-graduation impact, quality and safety, clinical audit, Australia

## Abstract

**Objectives:**

This study explores how medical graduates and their workplace supervisors perceive the value of a structured clinical audit program (CAP) undertaken during medical school.

**Methods:**

Medical students at the University of Notre Dame Fremantle complete a structured clinical audit program in their final year of medical school.  Semi-structured interviews were conducted with 12 Notre Dame graduates (who had all completed the CAP), and seven workplace supervisors (quality and safety staff and clinical supervisors).  Purposeful sampling was used to recruit participants and data were analysed using thematic analysis.

**Results:**

Both graduates and workplace supervisors perceived the CAP to be valuable. A major theme was that the CAP made a contribution to individual graduate’s medical practice, including improved knowledge in some areas of patient care as well as awareness of healthcare systems issues and preparedness to undertake scientifically rigorous quality improvement activities. Graduates perceived that as a result of the CAP, they were confident in undertaking a clinical audit after graduation.  Workplace supervisors perceived the value of the CAP beyond an educational experience and felt that the audits undertaken by students improved quality and safety of patient care.

**Conclusions:**

It is vital that health professionals, including medical graduates, be able to carry out quality and safety activities in the workplace. This study provides evidence that completing a structured clinical audit during medical school prepares graduates to undertake quality and safety activities upon workplace entry. Other health professional faculties may be interested in incorporating a similar program in their curricula.

## Introduction

Australia’s health care system has a reputation for providing high quality, publicly funded healthcare to the majority of the population.  To both maintain and improve this, it is important that doctors have the appropriate knowledge and skills to carry out safety and quality activities.  This is reflected in the Australian Medical Council’s accreditation standards which state that primary medical education programs must include  ‘a systems approach to improving the quality and safety of health care’ and ‘self-[evaluation of one’s] own professional practice’.^1^

The School of Medicine at the University of Notre Dame, Fremantle, Australia (Notre Dame), addresses evidence-based practice and quality and safety in medical practice throughout the four-year graduate curriculum, culminating in all final year students undertaking a supervised clinical audit.^2^ At the beginning of their final year, Notre Dame medical students receive instruction on audit techniques via a series of lectures and a detailed handbook which covers audit methodology.  Students may choose an audit topic from any discipline, but most select one from a list of clinical improvement priorities identified by participating health services.  Examples of previous topics include whether screening for gestational diabetes meets current guidelines, the management of post-partum hemorrhage, whether patients on surgical wards are being appropriately assessed for venous thromboembolism, and the appropriateness of prescribing specific antibiotics. Students are required to complete all steps of the audit process by first writing a proposal that includes aims and objectives, identifying an appropriate evidence-based SMART (specific, measurable, achievable, related and time-bound) standard relating to healthcare delivery and critically appraising the evidence supporting the chosen standard. Part of their proposal includes designing a data collection tool to assess whether the healthcare provided meets this standard. As per the normal procedures in clinical settings, students must obtain written approval from the relevant Clinical Quality and Safety Committee (or equivalent) of the health service where they plan to conduct their audit. A student peer-reviewer and academic staff formatively assess each student proposal and give detailed feedback to ensure validity and usefulness of the planned audit. Students then collect and analyse data, report their results back to workplace supervisors and formulate an action plan that addresses possible improvements. Audits are limited to 20-30 cases for an individual, or 30-40 cases for group (maximum of three students) audits. Assessment of the final written audit report contributes 10% of the total year summative assessment.  This report includes a section where students reflect on the experience of conducting the audit and what they have learnt from this.  To facilitate reflection, students are encouraged to keep a reflective blog throughout the time they are conducting the audit. 

A previous evaluation of the clinical audit program (CAP) found that overall student and supervisor satisfaction with the audit program was high, with more than 90% of supervisors reporting that the students’ audits were useful to the health service.^2^ In addition, a recent validation study of the CAP based on contemporary theories of assessment validity^3^^,^^4^ found that, from an assessment perspective, the CAP is meeting professional accreditation standards in preparing students for practice upon graduation and concluded that it is a valid and meaningful component of the assessment program for final year medical students.^5^  Studies in other settings have also shown positive educational outcomes of teaching clinical audit to medical students, and particularly engaging medical students in conducting research and clinical audit. For example, British medical students who participated in a national student audit and research collaboration developed higher levels of confidence and appreciation of academic principles and skills such as data collection,^6^ while a study at Dartmouth Medical School found that students learnt both about the subject matter of the area that they were auditing, whilst also absorbing the basics of continuous quality improvement.^7^ However, to truly assess the worth of any medical education initiative, it is necessary to look beyond its immediate educational outcomes to its longer-term effects, particularly on students after they graduate and are in the workplace.  To date, this has not been done for the Notre Dame CAP, and there appears to be a scarcity of evidence for the longer-term effects of similar programs teaching quality improvement in other medical schools.  The aim of this study was, therefore, to investigate whether the short-term educational outcomes of the Notre Dame CAP were translated into workplace practice. The primary research questions were:

     1. How do Notre Dame medical graduates (termed graduates) think the CAP has influenced their medical practice, including their involvement in quality improvement activities?  

     2. How do their workplace supervisors (those involved in quality and safety activities and clinical staff in teaching hospitals and general practices) think that completing the CAP affects graduates’ performance as junior doctors, including their involvement in quality improvement activities?  

A secondary objective was to identify potential areas for program improvement.

## Methods

### Study design and participants

This was a qualitative descriptive study. The study used a semi-structured interview technique for data collection and employed content analysis approach to analyse the data.  Interviews were conducted with Notre Dame medical graduates (who had undertaken the clinical audit whilst in medical school) and workplace supervisors, defined as quality and safety improvement officers/committee members or clinical supervisors at hospitals and general practices where Notre Dame graduates are employed as junior doctors.  These workplaces participate in the clinical audit program (CAP) and so many of the workplace supervisors also had a role in supervising projects conducted by students completing the CAP.

### Recruitment process

An invitation to participate in this study was emailed to all medical students on the Notre Dame alumni register who graduated between 2008 (when the CAP commenced) and 2013.  Workplace supervisors were identified with the help of CAP staff and invited to participate via email.  Purposeful sampling using a maximum variation sampling technique was used to ensure participation of graduates of different genders and years of post-graduation workplace experience, and of workplace supervisors from medical and quality and safety backgrounds in public and private health services. To obtain an information rich sample, additional graduates and workplace supervisors were recruited using snowball sampling.  Recruitment and interviewing ceased when data saturation was reached and no new themes were emerging.

### Participants

A total of 19 participants took part in the study, 12 graduates (eight males and four females) and seven workplace supervisors. Half of the graduates had completed the clinical audit program in 2012, with three having completed this in 2010 and one in each of 2008, 2009 and 2013. Ten of the graduates were currently working in hospitals (with six undertaking specialist training programs) and the other two were working in general practice.  All the seven workplace supervisors (four female and three male) held a current role in quality improvement in their workplace.  Five of the workplace supervisors were also past or current CAP clinical supervisors, four of these in hospitals and one in general practice.

### Ethical issues

Informed consent was obtained from all participants and it was explained that their participation in the study was completely voluntary.  To further ensure that there was no potential for coercion particularly when interviewing graduates, all interviews were conducted by members of the research team who had not held a position of direct authority over this group at any time.  This study was approved by the University of Notre Dame’s Human Research Ethics Committee. 

### Data collection methods

Two semi-structured questionnaires were developed, with one for graduates and the other for workplace supervisors (as defined above).  Two members of the research team (IO and SD) conducted telephone interviews in 2015 and 2016 due to the geographical dispersion of participants across Australia and overseas.

Interviews with graduates asked about their post-graduate involvement with quality activities and whether this had been influenced by completing the CAP, as well as how they thought completion of the program had influenced their patient care.   Interviews with workplace supervisors asked whether they had noticed any difference between graduates who had and had not completed the CAP in relation to patient care and involvement in quality and safety activities. Both graduates and workplace supervisors were asked to identify any improvements to the CAP.  Interviews lasted approximately 30 minutes. 

### Data analysis

Following a qualitative descriptive methodology, interview data were analyzed using content analysis approach.^8^ Interviews were either audio-recorded or detailed notes were taken, depending on participant preference. Both audio recordings and notes were transcribed.  Initially, each typed transcript was broken down into segments largely corresponding to the research objectives and data were then categorised according to themes developed both inductively and deductively. Themes were refined by repeated re-analysis of the data and discussion between members of the research team. The majority of primary coding was undertaken by one member of the research team (SD). To achieve increased validity, a sample of transcripts were independently coded by another member (CB) and results compared.  All analysis was done manually using word processing software.

## Results

Two main overarching themes emerged from the data.   The first was directly related to the research objectives and was the perceived effect of the CAP on the individual graduates’ medical practice including patient care, awareness of healthcare systems issues and preparedness to participate in quality improvement activities. The second overarching theme was the contribution of the student audits to improving quality and safety of patient care.  This was not directly related to the research objectives and emerged unexpectedly during analysis. Subthemes are grouped and described using pertinent quotes under these two overarching themes.

### Effects of the clinical audit program on individual practice

#### Patient care

All but two of the graduates reported that the CAP had influenced the individual care they provide to patients, either directly or indirectly. Several stated that this was related to knowing more about the best practice standard of their audit topic.  One example of this is a graduate who had conducted an audit on the quality of discharge summaries and found that this had improved the quality of their own discharge summaries and their overall communications with community care providers. Another example is a graduate whose referral patterns were based on knowledge of the importance of timeliness in angioplasty in patients having a myocardial infarction (MI), which he attributed to doing his audit on this topic.  He said that:

“I’ve had two patients present with MIs to my GP [surgery] instead of going to emergency. Instead of sending them to the regional or to the outer metro emergency, which I know doesn’t have an angioplasty unit, I have sent them directly to a tertiary hospital”. [Male, 2009 graduate, interview 4]

Two graduates discussed how the CAP had influenced their practice more broadly, in relation to ensuring they were making evidenced-based decisions for optimal patient care. As one put it: 

“Now I can actually go and look at best practice and think, hold on a minute, am I actually giving my patients the best practice that's available?” [Male, 2013 graduate, interview 3]

Another said the clinical audit made him realize the importance of ‘making active decisions’ as follows:

“.. make active decisions in medicine and not just blindly follow what feels right. So there needs to be a reason as to why you’re doing things". [Male, 2008 graduate, interview 2]

Of the two graduates who did not think completing the audit had influenced their clinical care, one thought that this was because the audit involved him ‘going through a whole lot of files, and didn’t change anything he did anyway’.  The other thought it was because she didn’t have a good understanding of the audit process at the time.  Interestingly this student did note that completion of the CAP had influenced her patient documentation in terms of being more specific, saying that:

“I think it’s definitely made me be a bit more studious with how I document things and clarifying questions of, well is that - making sure that there’s no grey area of what I mean versus what somebody else means versus what the standard dictionary term means”. [Female, 2010 graduate, interview 7]

All workplace supervisors found it difficult to state whether there were differences in patient care delivered by graduates who had or had not completed the CAP.  However, all said that this was either because they were not in a position to observe graduates’ clinical work (clinical and safety officers) and/or they were not aware of which doctors under their supervision had completed the program (clinical supervisors). Nevertheless, one hospital-based senior clinician workplace supervisor said she knew a few ‘outstanding individuals’ who had done the CAP and after graduation had gone on to implement and also advocate for best practice.  An example she cited was: 

“I saw them collect blood samples properly [for blood culture], and [asked them they why they’ve done it that way] then found out they did an audit on this”. [Female, hospital-based senior clinician, interview 15]

#### Systems issues

Several graduates discussed that as junior doctors their awareness of broader health systems and quality improvement issues was improved.  As one put it:

“…the bigger picture, the running of the unit that I work in….running of waits lists and overall protocols and devising ways to perform procedures in a safe way”. [Female, 2010 graduate, interview 7]

Many workplace supervisor interviews reflected these views.  One workplace supervisor related this to students becoming more aware of quality issues in general.  He said:

“… I think it would get the students in to realising that there is best practice medicine, quality practice, quality processes, and it's important to measure - to always be measuring or even having awareness of are we operating as well as we can?” [Male, GP supervisor, interview 8]

Another workplace supervisor thought the audit helped students become ‘work ready’ through an understanding of the medical workplace and particular barriers to effecting change.  He said:

“…understanding what the real medical workplace is like and also understanding how imperfect the real medical workplace is….. I've certainly had a lot of students comment on that, that in the end those things - they've learnt as much from that as they have from the actual topic that they studied.”  [Male, hospital-based senior clinician, interview 17]

#### Preparedness for, and participation in quality activities post-graduation

The skills and knowledge gained from the CAP were recurrent themes in graduate interviews, with all but one stating that completion of the CAP had improved their ability to participate in an audit post-graduation. Most stressed that having actually undertaken an audit as a student meant that they were familiar with the processes and had the ability and confidence required repeating this once in the workplace.  This is exemplified by the following quotes:

“I certainly think that having that start-to-finish process was quite useful and for us working in the hospital now, I know exactly what the process is I need to go through. I know who I need to speak to in ethics, and to get access to records, who I need to speak to. So it certainly makes it a lot easier to do simple audits”. [Female, 2010 graduate, interview 10]

“It had a monumental impact. We were completely equipped when we entered the workplace - well I certainly felt completely equipped when I entered the workplace to construct and complete a clinical audit with very little senior supervision”. [Male, 2013 graduate, interview 3]

The majority of workplace supervisors also remarked on the skills and knowledge that students gained from the audit.  As one stated:

“It certainly gets them to a level where they can participate and actually understand the audit process… it gives them an insight into the real world of hospitals, a clinical audit and how it works. So it certainly leaves them better equipped to participate in that, once they start working as junior doctors”. [Male, hospital-based senior clinician, interview 1]

Another workplace supervisor (hospital-based senior clinician) noted that some of the senior doctors they worked with did not have sufficient skills to conduct a clinical audit.   This was echoed by yet another workplace supervisor (quality and safety officer) who stated that she needed to ‘sit down and go through the whole step-by-step processes with doctors who had never previously done an audit.

The rigour of the program was seen as contributing both to student skills and knowledge, and the quality of the audits that students produced.  As one put it:

“The rigour associated with the program is such that they [the students] are forced to consider what variables they need to measure and how to measure these accurately”. [Female, hospital-based quality and safety officer, interview 19]

Interestingly, one workplace supervisor thought that the attention to rigour in methods could take away from students’ overall learning.  She stated:

“There is rigorous attention to scientific detail, so the audits are very focused on process.  This can lead to not enough focus on the interpretation of the results.  I want students to have a more composite overview – so what does it mean?” [Female, hospital-based senior clinician, interview 16]

For the same reasons as they found it difficult to comment on differences in patient care between graduates who had and had not done the CAP, most workplace supervisors also found it difficult to comment on whether completion of the program had influenced graduates level of participation in quality activities.  However, there was general agreement among workplace supervisors that conducting a clinical audit as a student would lead to greater awareness of and involvement in quality and safety activities post-graduation.  As one clinical safety manager explained:

“Those who have had clinical audit experience tend to be more interested in quality improvement activities and in sharing their ideas…..it’s hard to judge the impact of the audit, however from my role what I can see is that those MOs [medical officers] who have done audits before and learned about clinical auditing can collect data on and benchmark their own practice”. [Female hospital-based clinical quality and safety officer, interview 19]

Almost all graduates had undertaken quality improvement activities in their workplace with over one half having led or undertaken audits. Of note, one graduate conducted an audit as an intern on the same topic, using the tools he had developed as a medical student (in a different hospital with a different supervisor).  Time since graduation, and therefore opportunity to conduct an audit in the workplace, did not seem to influence whether graduates had done this, as all but one of those who graduated in 2013 had conducted an audit post-graduation while only one of those from pre-2010 had done so. Other related clinical activities that graduates had been involved in included sitting on standards settings committees, conducting literature reviews and attending morbidity and mortality meetings. 

#### Value realised in retrospect

Almost half of the graduates only realised the value of undertaking the CAP in retrospect. Several participants discussed negative feelings towards the program whilst they were undertaking it, due to the difficulty of the task and the high workload, particularly in the context of an overall stressful year of study.  As one put it:

“I feel like when I look back at the time I found it difficult but now I think it's a fantastic initiative and it's actually highly, highly beneficial”. [Male, 2010 graduate, interview 11]

This was further supported by some workplace supervisors who commented on the negative views some students had towards doing the clinical audit.  One linked this with the student’s stage of professional development:

“Of course the average medical student is still very ‘me’ focused. It's all about them. It's about getting their marks, passing their course, getting their degree…. There's no doubt doing that clinical audit contributes to their work readiness as junior doctors. They probably don't necessarily understand that while they're doing the audit, but I suspect it will dawn over the year”. [Male, hospital-based senior clinician, interview 1]

Workplace supervisors reported that topics should be of direct relevance to a junior doctor.  One female senior clinician related that students ‘get excited’ and engage more with the process when they audited a relevant topic that would be of use to them as a junior doctor.

### Contribution of the student audits to improving quality and safety of patient care

Although not directly asked of participants, the value of the CAP to the workplace was a recurring theme among workplace supervisor interviews with nearly all reporting changes in clinical practice at their workplace as a result of student audits.  Several workplace supervisors linked this to the process where students fed back to the unit or practice where the audit had occurred, raising awareness both of the unit’s performance and also the specific standard or guideline that constitutes best practice treatment.  One workplace supervisor described the students’ audits as:

“...incredibly helpful for the work I’m doing as the topics are very targeted to fit in around this and I’ve referenced their audit data in a number of settings.  It’s not normally what people would call research however it helps influence practice locally”.  [Female, hospital-based senior clinician, interview 15]

This same clinician also noted that the requirement that medical students present the information to the health service was very useful in terms of encouraging best practice as:

“if you [a senior clinician] present your analysis the clinical teams get their hackles up, but if medical students present it as ‘best practice’ then that’s a good way to bring about change”. [Female, hospital-based senior clinician, interview 15]

While graduates did not speak directly about the value of their audit to the workplace, one did reference this indirectly through how he had found participation in the program personally rewarding.  As he put it: 

“It makes me feel fulfilled, seeing that I've contributed to the health system in that way, even though some people may poo and go well, you didn't actually help a specific patient. Well, no I didn't. But when you look at a lot of the public health issues and everything else, you don't just help people by physically treating them”.  [Male, 2013 graduate, interview 3]

## Discussion

Our findings indicate that graduates believe the Notre Dame CAP enables them to deliver better patient care as soon as they enter the medical workforce. Through conducting all steps of a clinical audit, in a real medical workplace, students developed an appreciation for the importance of delivering evidence-based best practice care to individual patients.  In some cases, the in-depth knowledge they gained about a specific topic directly improved the care they provided to patients. By examining the process by which care is delivered, and looking at ways to improve this, students gained an appreciation of the systemic issues that contribute to individual outcomes within the health system.  Students who completed the reported graduating with skills and knowledge in clinical audit and quality improvement which they translated into practice after graduation enabling them to measure and improve their own and others’ practice.  Previous studies have shown that using clinical audit as a teaching tool for both medical students and residents can lead to short-term improvements in patient care^9^^-^^11^ and educational outcomes.^12^^-^^14^  The results reported in this paper support these findings by demonstrating that undertaking a scientifically valid and academically rigorous clinical audit in medical school can improve practice post-graduation. 

This study found that graduates reported that the CAP had equipped them with the skills, knowledge and confidence that they need to conduct a clinical audit in their workplace upon graduation.  In terms of what contributed to this outcome, both graduates and workplace supervisors stressed the experience of actually having completed an audit during medical school. This is consistent with the principle espoused in Miller’s pyramid which argues that tasks that assess what the student does (action) in a real clinical setting are much better at predicting their future behaviour than those that assess knowledge, competence or performance.^15^   Although learning by experience is widely used within medical education,^16^ there are aspects of the CAP which may increase post-graduation effects. These include the requirements for students to work in consultation with workplace supervisors to develop an implementation plan based on their audit findings and in their summative assessment to write a reflection on what they learned from their experience of developing and completing an audit. This grounds the program in an experiential learning framework, a process which emphasises learning through reflection on new experiences^17^ and which has been shown to be related to graduate preparedness for the workplace.^18^

Both workplace supervisors and graduates valued the CAP.  The graduates focused on the educational value of the program, and while workplace supervisors acknowledged this, they also valued the program for its ability to improve patient care as a direct result of audits conducted by students. That this theme emerged so strongly from our data was unexpected, as exploring this aspect of the CAP was not an aim of this project.  However the capacity for medical students undertaking clinical audit to achieve both educational and patient improvement outcomes has been noted previously,^6^^,^^19^ and the dual educational and patient improvement outcomes was a focus of a recent systematic review^20^  that examined factors leading to success in teaching quality improvement to medical students and resident (post graduate trainee) doctors. This review presented a conceptual model that puts the learner at the centre of two ‘overlapping worlds’ of the clinical workplace and the education system.  While this contributes greatly to the high value of teaching clinical audit to medical students, it also shows the potential for conflict as the clinical workplace and the university may at times have different aims, priorities and needs.  In the results reported in this paper, this potential was illustrated by participants’ feedback that some processes and differing expectations from the clinical workplace and the university were perceived as being problematic.  From the university perspective, any task for assessment must adhere to exacting standards and fit within the overall curriculum, whereas the workplace emphasis will be on using findings to improve patient care. Most students will naturally focus more on fulfilling assessment criteria, and this may explain the comment by one supervisor who felt the rigour of the audit resulted in some students being more focused more on academic rigour and process than the implementation of quality improvement.

It may not be possible to completely reconcile the differing needs of the university and clinical workplace. Nonetheless, this project provided insight into areas to consider for improvement.  One of these is choice of audit topics.  While topics already go through a process to determine their suitability, more discussion could take place with health services to ensure that topics will be of immediate usefulness to the medical students upon graduation. This may also increase the perceived relevance and therefore engagement and learning of students undertaking the CAP at the time they are doing this, in line with adult learning principles that professionals learn best when they can see the need for learning.^21^

The finding that some medical students may view clinical audit negatively when undertaking this has been shown in previous work.^22^  However, practitioners’ active participation in quality improvement is key in providing safe health care.  Moreover, the ability to conduct clinical audit is an expectation of the Australian Curriculum Framework for Junior Doctors^23^ and most Australian specialities which incorporate clinical audit and quality improvement into their training programs and ongoing professional development requirements.^24^^,^^25^  It is therefore vital that the requisite skills, attitudes and knowledge for quality improvement be taught and assessed in primary medical education curricula.  Our results show that graduates do ultimately recognise and appreciate this component of their undergraduate curriculum. More broadly this finding illustrates an important issue for medical educators; the purpose of medical school is to produce competent and safe doctors, therefore evaluations of medical school curricula need to consider graduate performance as their ultimate outcome measure.  Medical students cannot be expected to know what they need to function in the workplace, so curriculum developers always need to consider the post-graduation impacts rather than making curriculum changes based on satisfaction ratings of students who have just finished a particular course or subject. 

Limitations of the study include the potential for bias in the sample. There was a relative lack of graduates from earlier years of the program, most likely due to contact details in the alumni database being less up to date for this group.  There were also a lower number of females in the sample, which is not reflective of the graduate mix where females generally comprise more than 50% of the graduating class at Notre Dame.  However, analysis by comparing subgroups found no systematic differences in interview results between those who graduated in earlier and later years, and males and females. Workplace supervisors’ observations of CAP graduates were limited because they generally did not know which graduates had or had not completed the program.  Those the researchers believed would have more insight into these issues were actively recruited.  Ultimately, however, it was not possible to obtain sufficient data on the differences between graduates who had and had not undertaken the CAP. Finally, it is possible that respondents in this study had a more positive view of the program or stronger views on the program, and this motivated them to take part in this study.  The diversity of viewpoints captured in the sample provides some confidence in the findings.  Future research may focus on capturing an objective comparison of specified patient care outcomes and/or involvement in quality improvement activities with a control group cared for by graduates who had not undertaken the CAP.  However, the lack of an appropriate control group and the potential confounders involved would make this research challenging. 

Despite the stated limitations, the qualitative design of our study allowed an in-depth view of the longer term effects of the CAP, a clearer understanding of the aspects of the program that contribute to its success, and identified areas for improvement.

## Conclusions

Most program graduates interviewed for this study reported that the CAP provided them with the attitudes, skills and knowledge to conduct quality improvement activities as health professionals, as well as improving their individual patient care. While some workplace supervisors expressed similar views, they focused more on the value of the audits, which allow medical students to make a meaningful contribution to patient care. 

The Notre Dame CAP has been running since 2008. This study provides some evidence that this is a sustainable program that provides lasting value for both graduates and the health services in which they work. Other medical schools may be interested in incorporating this or similar programs in quality improvement into their curricula that allow students to make a meaningful contribution to patient care whilst acquiring indispensable educational outcomes.  Similar programs could also be used by other health teaching faculties such as nursing and allied health professions. 

### Acknowledgements

Thanks to Notre Dame, Fremantle staff involved in CAP teaching and assessment, and audit supervisors and clinical quality and safety staff at health services where students undertake their audits, as well as to all participants who agreed to be interviewed for this study.

This research was partially supported by a Citation Award from the Australian Government's Office of Learning and Teaching.

### Conflict of Interest

The authors declare that they have no conflict of interest.
